# Supplementary figures and images for: Genetic structure and designing a preliminary core collection of Zizania latifolia in China based on 12 microsatellites markers
Source: PeerJ. 2025 Feb 21;13:e18909. doi: 10.7717/peerj.18909 (PMC11849519; doi:10.7717/peerj.18909)

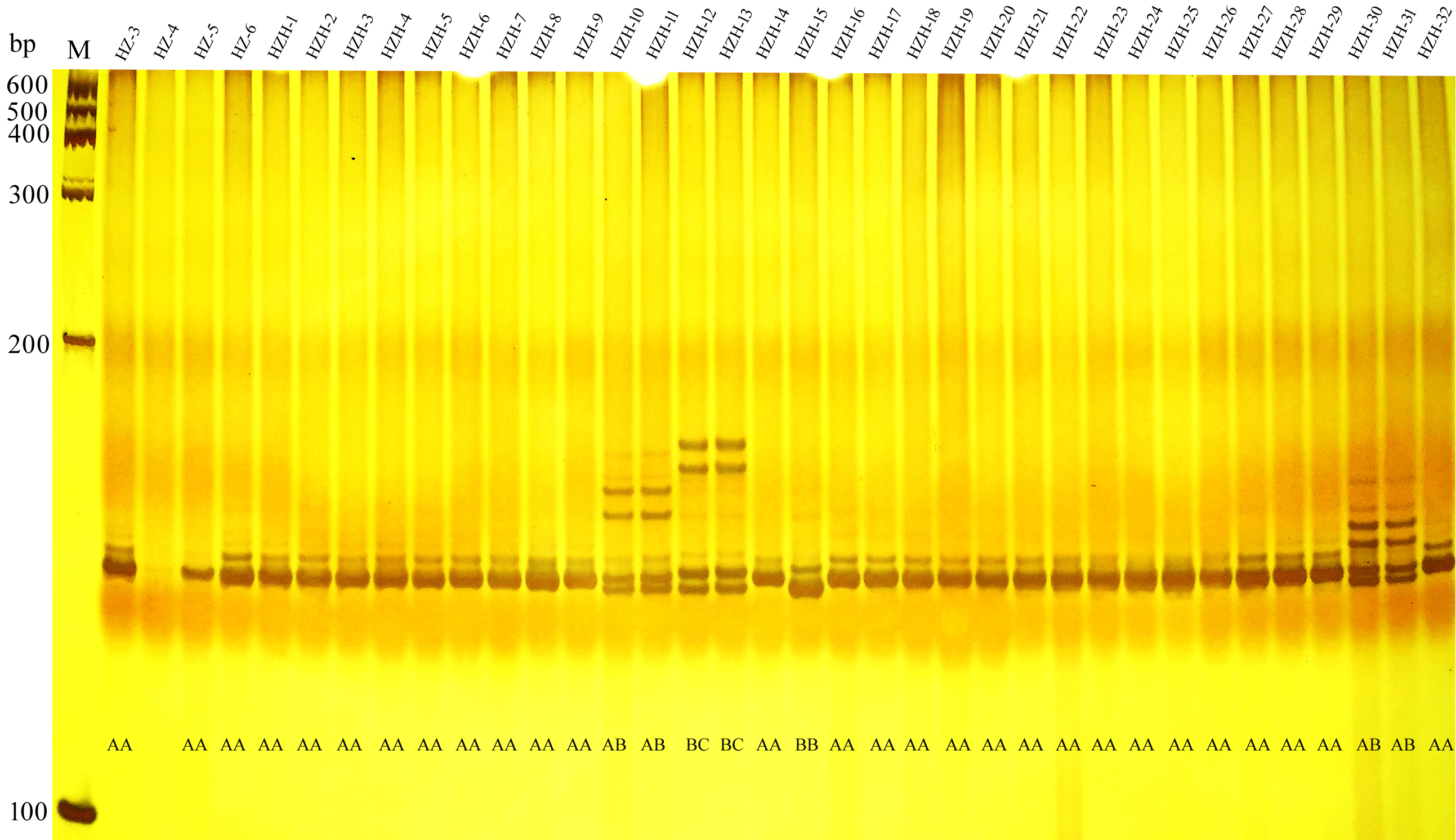

Supplement: Supplemental Information 1 — The individual code and corresponding co-dominant genotype were illustrated for each lane. M indicated the 100 bp ladder Marker. [file peerj-13-18909-s001.png]

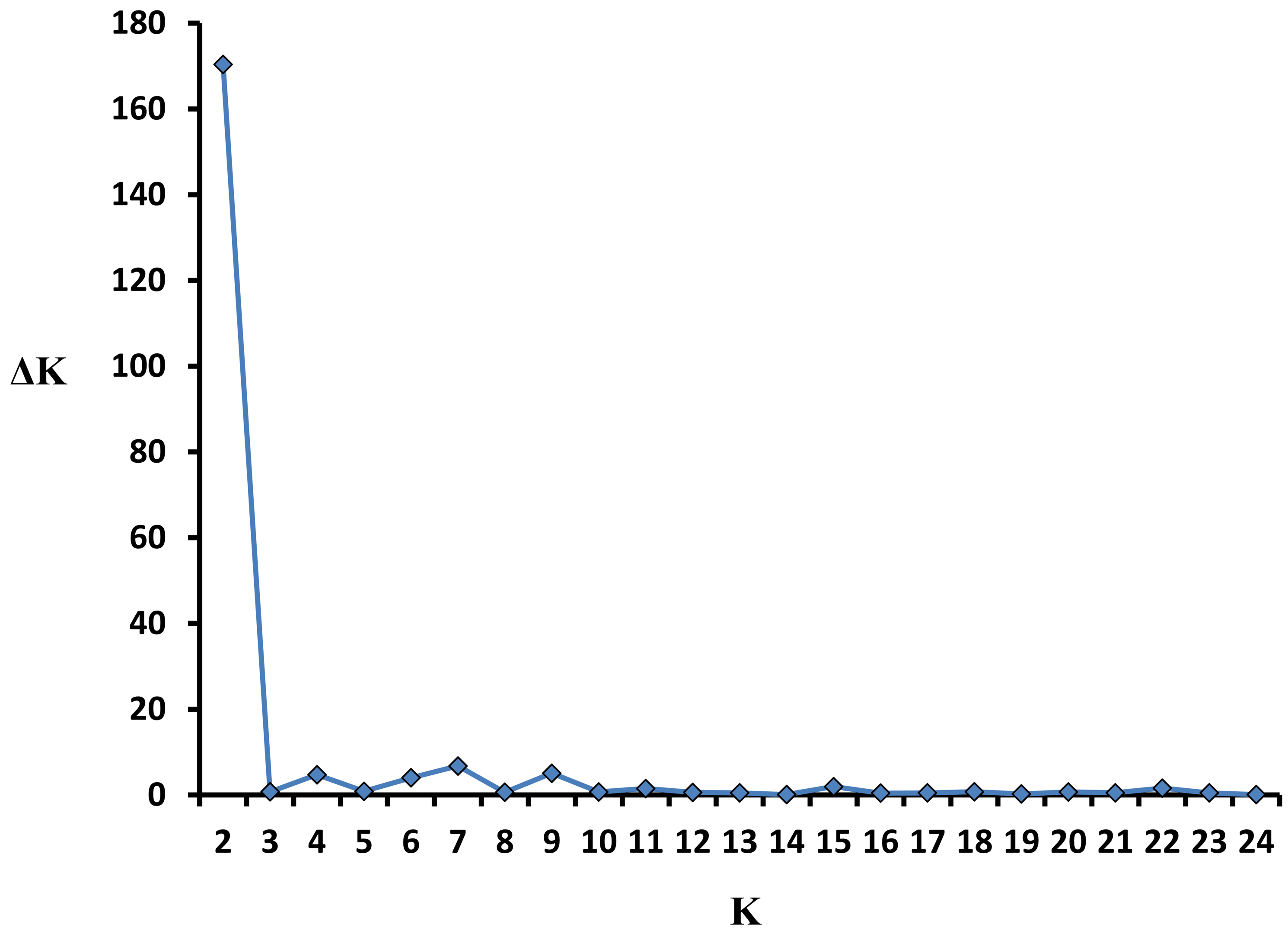

Supplement: Supplemental Information 2 — Calculated according to Evanno et al. (2005) for 10 simulations in the STRUCTURE program. [file peerj-13-18909-s002.png]

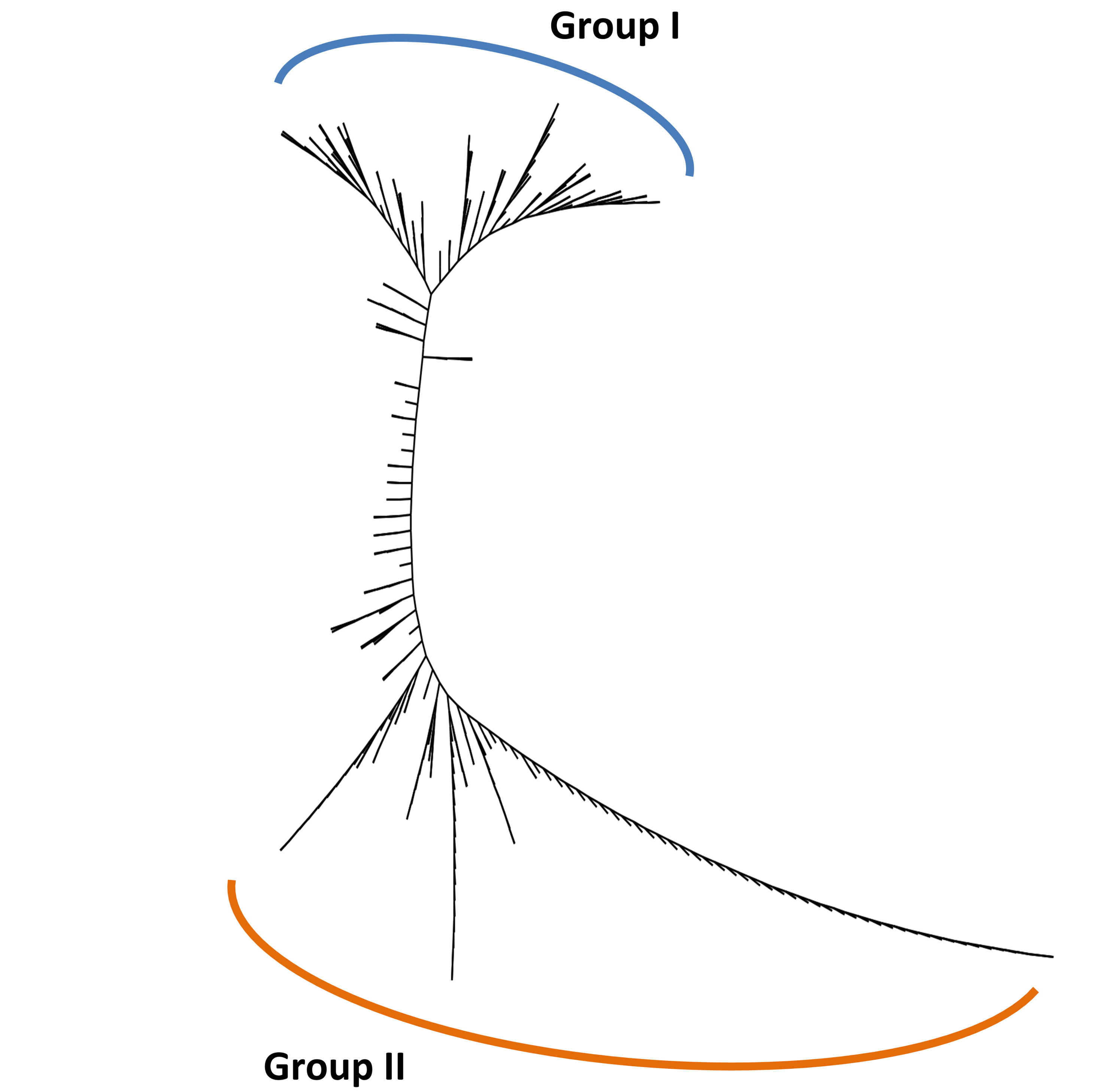

Supplement: Supplemental Information 3 [file peerj-13-18909-s003.png]
