# Supplementary material for: Genetic structure and designing a preliminary core collection of Zizania latifolia in China based on 12 microsatellites markers
Source: PeerJ. 2025 Feb 21;13:e18909. doi: 10.7717/peerj.18909 (PMC11849519; doi:10.7717/peerj.18909)
Supplement: Supplemental Information 4 [file peerj-13-18909-s004.docx]

**Table S1 Descriptive statistics of the 12 SSR markers scored on 357 wild *Z. latifolia* accessions.**

| **Locus** | ***N*_a_** | ***A*_e_** | ***I*** | ***H*_o_** | ***H*_e_** | ***PIC*** | ***F*_st_** |
| --- | --- | --- | --- | --- | --- | --- | --- |
| **ZM4** | 2.240 | 1.657 | 0.533 | 0.007 | 0.338 | 0.484 | 0.348 |
| **ZM5** | 1.600 | 1.322 | 0.291 | 0.000 | 0.198 | 0.384 | 0.615 |
| **ZM13** | 1.560 | 1.317 | 0.284 | 0.017 | 0.196 | 0.378 | 0.615 |
| **ZM16** | 1.880 | 1.389 | 0.329 | 0.000 | 0.194 | 0.323 | 0.382 |
| **ZM24** | 1.800 | 1.387 | 0.343 | 0.000 | 0.221 | 0.358 | 0.483 |
| **ZM25** | 1.120 | 1.040 | 0.045 | 0.000 | 0.028 | 0.033 | 0.189 |
| **ZM26** | 1.840 | 1.489 | 0.347 | 0.000 | 0.207 | 0.473 | 0.577 |
| **ZM28** | 1.520 | 1.174 | 0.180 | 0.000 | 0.106 | 0.157 | 0.193 |
| **ZM30** | 2.240 | 1.685 | 0.513 | 0.101 | 0.313 | 0.491 | 0.428 |
| **ZM35** | 1.640 | 1.329 | 0.274 | 0.000 | 0.176 | 0.390 | 0.650 |
| **ZM44** | 2.640 | 2.055 | 0.642 | 0.000 | 0.373 | 0.715 | 0.472 |
| **Zt23** | 1.800 | 1.476 | 0.365 | 0.015 | 0.237 | 0.409 | 0.468 |
| **Mean** | 1.823 | 1.443 | 0.345 | 0.012 | 0.207 | 0.383 | 0.452 |

*N_a_*, mean number of alleles; *A*_e_, number of effective alleles; *I*, Shannon’s Index; *H*_o_, observed heterozygosity; *H*_e_, expected heterozygosity; *PIC*, Polymorphism Information Content; *F*_st_*,* genetic differentiation coefficient.
