# Supplementary material for: Genetic structure and designing a preliminary core collection of Zizania latifolia in China based on 12 microsatellites markers
Source: PeerJ. 2025 Feb 21;13:e18909. doi: 10.7717/peerj.18909 (PMC11849519; doi:10.7717/peerj.18909)
Supplement: Supplemental Information 5 [file peerj-13-18909-s005.docx]

**Table S2 Analysis of Molecular Variation (AMOVA) for *Z. latifolia***

| **Source of variation** | **d.f.** | **Sum of squares** | **Variance components** | **Percentage of variation (%)** |
| --- | --- | --- | --- | --- |
| Among populations | 24 | 836.96 | 1.11 | 37.11 |
| Within populations | 332 | 1244.37 | 1.87 | 62.89 |
| Total | 356 | 2081.33 | 2.98 |  |
